# Pacman in the sky with shadows: the effect of cast shadows on the perceptual completion of occluded figures by chimpanzees and humans

**DOI:** 10.1186/1744-9081-6-38

**Published:** 2010-07-08

**Authors:** Masaki Tomonaga, Tomoko Imura

**Affiliations:** 1 Primate Research Institute, Kyoto University, Inuyama, Aichi 484-8506, Japan

## Abstract

**Background:**

Humans readily perceive whole shapes as intact when some portions of these shapes are occluded by another object. This type of amodal completion has also been widely reported among nonhuman animals and is related to pictorial depth perception. However, the effect of a cast shadow, a critical pictorial-depth cue for amodal completion has been investigated only rarely from the comparative-cognitive perspective. In the present study, we examined this effect in chimpanzees and humans.

**Results:**

Chimpanzees were slower in responding to a Pacman target with an occluding square than to the control condition, suggesting that participants perceptually completed the whole circle. When a cast shadow was added to the square, amodal completion occurred in both species. On the other hand, however, critical differences between the species emerged when the cast shadow was added to the Pacman figure, implying that Pacman was in the sky casting a shadow on the square. The cast shadow prevented, to a significant extent, compulsory amodal completion in humans, but had no effect on chimpanzees.

**Conclusion:**

These results suggest that cast shadows played a critical role in enabling humans to infer the spatial relationship between Pacman and the square. For chimpanzees, however, a cast shadow may be perceived as another "object". A limited role for cast shadows in the perception of pictorial depth has also been reported with respect to human cognitive development. Further studies on nonhuman primates using a comparative-developmental perspective will clarify the evolutionary origin of the role of cast shadows in visual perception.

## Background

**Figure 1:**
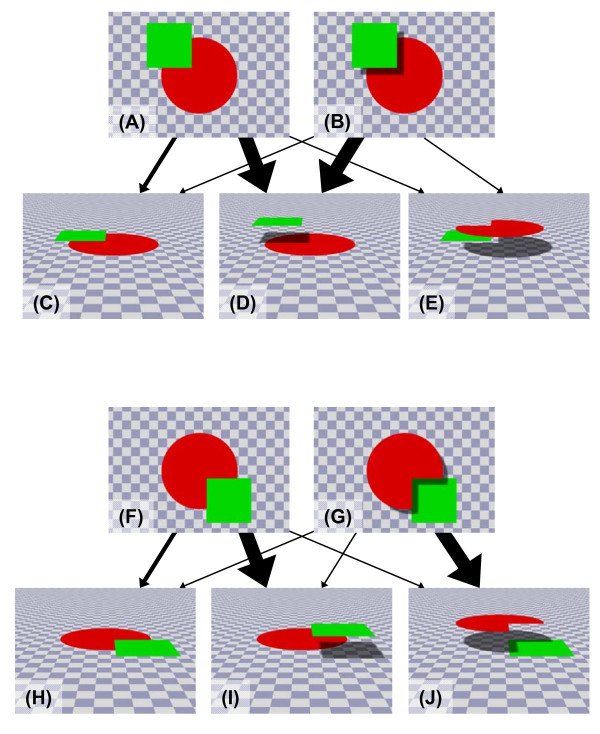
**Amodal completion and pictorial depth perception**. Thickness of arrows indicates the plausibility of the percept implied by stimuli A, B, F, and G. For humans, the cast shadow determined the spatial relationship between the occluder and Pacman (or full circle) in a relatively unambiguous fashion.

Humans readily perceive whole shapes as intact when portions of these shapes are occluded by another object (see Figures. 1A and 1F). For example, when we see the objects depicted in Figure 1A, we do not recognize these as a red Pacman and a green square but rather see them as a red full circle occluded by a green square. This phenomenon is often called perceptual completion. According to Kanizsa [1], perceptual completion can be categorized into modal completion, including completion involving subjective contours, and amodal completion, as shown in Figures 1A and 1F. Like modal completion [e.g., [2,3]], amodal completion has been observed not only in human adults, but also in human infants [e.g., [4]], nonhuman primates [5-8], and some species of birds [9,10]. Sato et al. [5] trained one adult female chimpanzee on a matching-to-sample task in which unitary and separated rectangles appeared as stimuli. After training, the chimpanzee was tested with a sample stimulus partly occluded by a large object. Although the sample rectangles were physically separated by this occluder, they would be perceived as unitary if perceptual completion occurred. The results clearly indicated that the chimpanzee achieved perceptual completion; that is, she consistently chose the unitary rectangular stimulus. In the context of these and other results, it would appear that amodal completion is a quite basic visual-perceptual function for visual animals [cf. [11]], although pigeons have exhibited exceptionally negative results[[7,12-16], but see [17,18] for positive results in pigeons]. For example, Fujita and Ushitani [14] tested pigeons and humans under a visual search task in which the target was a Pacman-like lozenge with or without an occluder, and the distractors were intact lozenges. The response times of humans increased significantly over those obtained under other conditions when some portion of the lozenge target was occluded by another square, showing strong amodal completion. In contrast, however, pigeons demonstrated no such increase in response times, suggesting the absence of amodal completion.

Amodal completion is affected by many factors [19], such as (common) motion, regularity of edges (good continuation or so-called relatability, [20]), and various kinds of pictorial depth cues. Motion and regularity cues have been examined extensively in nonhuman primates [e.g., [5,8,11]], although the role of pictorial depth information, such as T-junctions [7,21], linear perspectives [[22], cf. [23,24]], and shadows [cf. [24]] has received relatively less attention from comparative-cognitive researchers. When humans see occluded figures, we infer the three-dimensional spatial relationship between the figure and the occluder (e.g., the occluder superimposed on the figure, as shown in Figure 1D) in addition to completing the whole shape. Working within the context of scarce comparative research in this domain, Fagot et al. [22] tested the effects of linear perspectives on the amodal completion of occluded figures by baboons. Baboons did not show amodal completion in their previous experiments [6]. When the occluded shape was presented on a linear-perspective background, however, the baboons perceived a Pacman-like occluded circle as a full circle [22]. Their results may suggest that additional perspective cues facilitated the depth perception implied by T-junctions.

The other, less focal, depth cue is shadow information. For example, when we see a picture such as Figure 1B, in which a shadow is cast by a green occluder on a red Pacman, we readily recognize the spatial relationship between these objects and complete the occluded object (the occluder in the sky and a full circle, not a Pacman, on the ground, as in Figure 1D). Other interpretations, such as those depicted in Figures 1C (Pacman and green square on the same ground) and 1E (Pacman floating in the sky), seem implausible. Indeed, cast shadows provide information about the three-dimensional spatial arrangement of objects and background. In addition, the compulsory amodal completion in Figure 1G would have been interrupted and we would have recognized that the Pacman figure in the sky was casting a shadow cast on the green square as soon as the darker area adjacent to Pacman was recognized as a cast shadow. When the cast shadow is removed, as in Figure 1F, we easily complete the occluded portion and perceive the stimulus depicted in Figure 1I, a green square floating above and occluding the full red circle.

**Figure 2:**
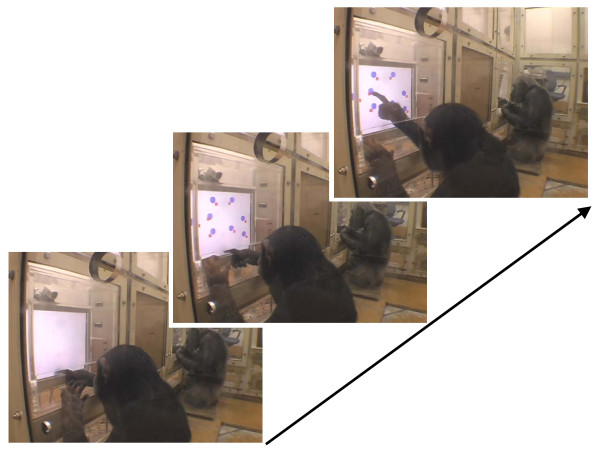
**A young female chimpanzee, Cleo, performing the visual search task in Experiment 1**.

Perception of shadows by nonhuman animals has not been studied extensively from a comparative perspective. A few studies on the perception of the shading and brightness gradient of the surface of an object have been conducted in birds [25,26] and chimpanzees [27,28]. However, quite a few studies on the perception of cast shadows in nonhuman animals have been conducted [24]. Imura and Tomonaga [24] examined the effects of cast shadows on chimpanzees' perceptions of size constancy in the context of a linear perspective [23]. In this context, the size of an object located near the vanishing point is relatively overestimated, an effect referred to as the "corridor illusion" [29,30]. Imura and Tomonaga examined the role of additional three-dimensional depth information derived from shadows cast on objects. However, the effect of cast shadows was rather limited in chimpanzees, and their performances were modified only when the cast shadows were moving along with moving objects. Moving cast shadows also affected the perception of motion trajectories in Japanese macaque infants [31], chimpanzees [31], and human infants [[32], cf. [33]]. To our knowledge, however, no studies on the effect of cast shadows on amodal completion in human and nonhuman animals have been conducted. The present study examined this effect in chimpanzees and humans from a comparative-cognitive perspective using a visual search task (see Figure 2) similar to that used with pigeons [14]. Chimpanzees were tested with no-shadow stimuli to replicate previous studies [5] in the context of different task demands, and we further examined the role of cast shadows in amodal completion. On the basis of previous findings, we hypothesized that the chimpanzees would readily complete the partially occluded objects, but that, chimpanzee's perception of the spatial relationship between objects and occluders would not be strongly modified by the cast shadows.

## Results

### Experiment 1: Visual search for Pacman

The first experiment was conducted to replicate previous findings of object unity or amodal completion in chimpanzees [5] engaged in a visual search task similar to that used in experiments with birds [14].

**Figure 3:**
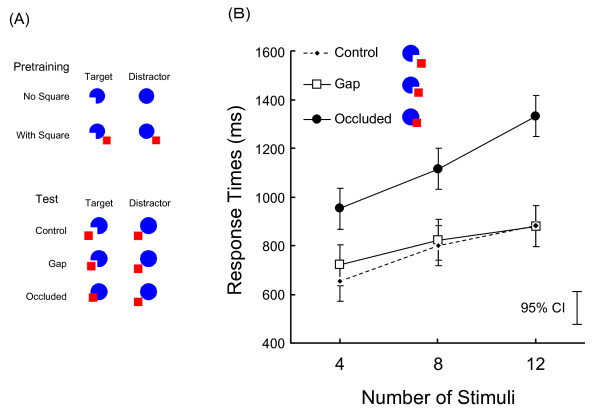
**Summary of Experiment 1. **(A) Stimuli used in Experiment 1. (B) Mean response times on correct trials under each condition in Experiment 1. Error bars indicate the 95% confidence intervals (CIs) of the differences in means.

All four chimpanzee participants performed very accurately in the initial training (99.6% correct responses on average) and test sessions [1.1%, 1.7%, and 7.4% errors under control, gap, and occluded trials, respectively (Figure 3A)], although a significant difference in Pacman-square distance [general linear mixed model analysis, *F*(2, 112) = 16.42, *p *< 0.001] emerged. Figure 3B shows the mean response times on correct trials under each condition averaged across participants. Error bars show 95% CIs for the differences between means. Chimpanzees showed longer response times when the target was a circle with a square (occluded trials) than under the control and gap trials. Mixed model analyses revealed significant main effects of number of stimuli [*F*(2, 112) = 69.15, *p *< 0.001] and Pacman-square distance [*F*(2, 112) = 29.02, *p *< 0.001], but no significant interaction [*F*(4, 112) = 2.128, *p *= 0.082].

### Experiment 2: Effects of cast shadows on visual search for Pacman

**Figure 4:**
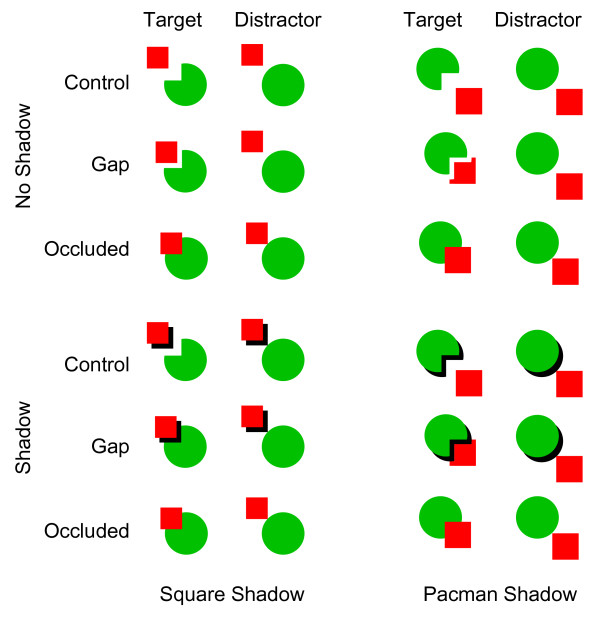
**Stimuli used in Experiment 2**. Note that the gap width depicted in this figure is exaggerated rather than drawn to scale.

Experiment 2 added cast shadows to the square or Pacman (see Figure 4). The same four chimpanzees and an additional eight humans participated in this experiment. The error rates were 4.6% and 0.1% for chimpanzees (see Table 1) and humans, respectively. Thus, we did not conduct any statistical analysis with human accuracy data. The mixed-model analyses conducted on the accuracy data under square-shadow condition in chimpanzees found significant main effects for shadow [*F*(1,114) = 7.05, *p *= 0.009] and Pacman-square distance [*F*(2,114) = 8.36, *p *< 0.001], but no two-way interaction [*F*(2,114) = 2.02, *p *= 0.138] was observed (Table 1). *Post-hoc *comparisons among the Pacman-square distances on the basis of estimated marginal means confirmed that performances under the control trials were better than those under the gap (*p *= 0.037) and occluded (*p *< 0.001) trials. Under the Pacman-shadow condition the effect of shadow was significant [*F*(1,114) = 16.66, *p *< 0.001], and the effect of Pacman-square distance was marginally significant [*F*(2,114) = 3.02, *p *= 0.053], but the two-way interaction was not significant [*F*(2,114) = 0.17, *p *= 0.841].

**Table 1:** Percentages of errors (and standard errors) under each condition among chimpanzees in Experiment 2.

		Control	Gap	Occluded
Square-Shadow	No Shadow	0(0.0)	1.3(1.5)	7.2(4.5)
	Shadow	1.3(0.9)	10(5.6)	10(5.2)
Pacman-Shadow	No Shadow	0(0.0)	1.3(1.5)	3.4(2.5)
	Shadow	4.4(2.3)	7.5(1.3)	8.8(2.9)

**Figure 5:**
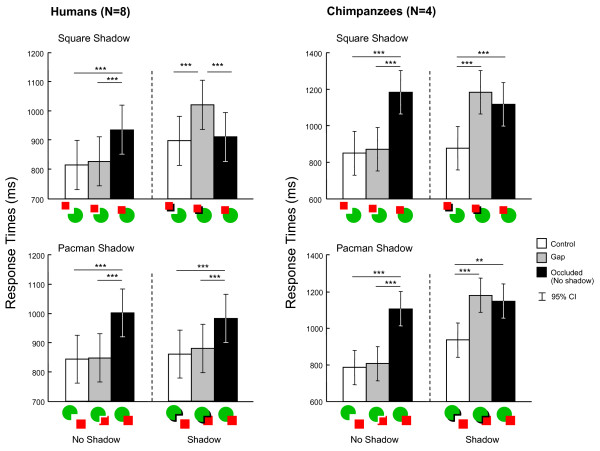
**Mean response times on correct trials under each condition in Experiment 2**. Error bars indicate the 95% CIs in the differences in means. **: *p *< 0.01, ***: *p *< 0.001.

Figure 5 shows the mean response times on correct trials under each condition for each species. All participants responded slowly under the occluded trials (without cast shadows) and rapidly under the control trials, replicating the previous results. The response times under the gap trials, however, varied between species and across conditions. The shadow cast by Pacman on the square prevented perceptual completion in humans but not in chimpanzees.

Mixed model analyses on the response-time data obtained from humans found significant main effects and the two-way interaction under the square-shadow condition [shadow, *F*(1,75) = 26.51, *p *< 0.001; Pacman-square distance, *F*(2,75) = 7.55, *p *= 0.001; interaction, *F*(2,75) = 14.87, *p *< 0.001]. *Post-hoc *analyses revealed that the response times under the occluded trials were significantly longer than were those under the control and gap trials (*p*s < 0.001) in the context of the no-shadow condition, whereas the response times under the gap trials were significantly longer than were those under the control and occluded trials (*p*s < 0.001) in the context of shadow condition. Under the Pacman-shadow condition, only the main effect of Pacman-square distance was significant [*F*(2,75) = 18.77, *p *< 0.001; shadow, *F*(1,75) = 0.226, *p *= 0.636; interaction, *F*(2,75) = 0.547, *p *= 0.581]. *Post-hoc *analyses revealed that the response times under the occluded trials were significantly slower than were those under the control and gap trials (*p*s < 0.001).

For chimpanzees, both main effects of shadow [*F*(1,95) = 5.90, *p *= 0.017] and Pacman-square distance [*F*(2,95) = 19.97, *p *< 0.001] and a two-way interaction [*F*(2,95) = 9.38, *p *< 0.001] under the square-shadow condition. *Post-hoc *comparisons confirmed that the chimpanzees showed significantly longer response times under the occluded trials than under the control and gap trials (*p *< 0.001) in the context of the no-shadow condition, whereas they showed significantly faster response times under the control than under the gap and occluded trials in the context of the shadow condition (*p *< 0.001). All main effects and two-way interactions were significant under the Pacman-shadow condition [shadow, *F*(1,95) = 29.57, *p *< 0.001; Pacman-square distance, *F*(2,95) = 19.74, *p *< 0.001; interaction, *F*(2,95) = 7.89, *p *= 0.001]. *Post-hoc *comparisons revealed patterns of response times similar to those observed under the square-shadow condition. In the context of the no-shadow condition, the chimpanzees showed significantly longer response times under the occluded trials than under the control and gap conditions (*p *< 0.001), whereas they showed significantly faster response times under the control trials than under the gap (*p *< 0.001) and occluded (*p *= 0.002) trials in the context of the shadow condition.

## Discussion

The present study explored the effect of the shadows cast by figures or occluders on amodal completion by chimpanzees and humans. Experiment 1 replicated the finding of amodal completion in chimpanzees. These results were consistent with those of previous experiments with chimpanzees and capuchin monkeys engaging in a matching-to-sample task [5,8] and inconsistent with a series of studies in pigeons [7,12,14,16][but see [17,18] and baboons [6].

On the basis of the results of Experiment 1, the next experiment examined the effect of cast shadows. In general, when the shadow of the occluding square was cast on the Pacman figure, both species demonstrated perceptual completion. Figures with cast shadows were associated with stronger completion performances than were no-shadow figures, especially in humans. When a shadow was added to Pacman and cast on the square and background (Pacman-shadow condition), clear species differences emerged. Humans exhibited faster response times under this condition than under the no-shadow occlusion condition, whereas chimpanzees exhibited quite similar patterns of results under both conditions; that is, even when the shadow was added to Pacman, response times remained as long as those demonstrated under the no-shadow occlusion condition. These contrasting results suggest that these species processed the portions obscured by cast shadows (black areas) differently. Humans most likely recognized these black areas as shadows cast by the square or Pacman on the other object. Therefore, completion was enhanced when the shadow was added to the occluding square (implying it was located "on" the full red circle), whereas completion was disrupted by the cast shadow attached to the edge of Pacman (implying that Pacman was in the sky with shadows). For chimpanzees, on the other hand, the black area functioned as the other "object." If the black "object" were superimposed on Pacman in addition to the square, the results would have been the same as those obtained when this object was not presented. However, if the object were superimposed on the square, this object would have been integrated with the square, not with Pacman's shadow, and perceptual completion would have been achieved.

For both species, adding the cast shadow significantly increased the response times during the control trials in some conditions (i.e., square-shadow condition in humans and Pacman-shadow condition in the chimpanzees; *Post-hoc *analyses, *p*s < 0.001). These results indicate that an additional black area generally disrupted the participant's performances, but that obviously this effect alone could not explain the overall results.

## Limitation

The limited impact of cast shadows on perceptual completion in chimpanzees is reminiscent of the rather limited role of static cast shadows on the perception of pictorial depth [24]. The effect of the spatial relationship between the cast shadow and the object on size-constancy discrimination was evident only when the shadow and object moved in synchrony. For chimpanzees, cast shadows play a relatively limited role in pictorial depth perception. When we move the square or Pacman synchronously with their cast shadows, chimpanzees obtain results similar to those obtained in humans under the static condition, as in the present study.

The other possibility with respect to humans would involve the cues derived from T-junctions [21]. Detailed views of the target stimuli under gap and occluded trials reveal that the T-junctions remained unchanged for targets with and without shadows; that is, they were the contours of the square or cast shadow intersecting with the contour of the occluded circle. On the other hand, however, when a cast shadow was added to Pacman, the contour of the shadow intersected with the contour of the square in a reverse direction. Although this T-junction was quite subtle given that the width of the cast shadow was 1.3 mm (10% of the width of the occluder and 5% of the diameter of the circle), the interaction between T-junctions and cast shadows should be examined further in the future.

## Conclusion

In the current study, we clearly demonstrated species differences in the effect of cast shadows on perceptual completion. Compulsory amodal completion can be modified by cast shadows in humans, whereas cast shadows may be processed as a separate object rather than as a shadow by chimpanzees. Static presentation of cast shadows may affect the performances of chimpanzees. Interestingly, this disadvantage of static cast shadows has also been reported in studies with humans in a developmental-cognitive context [[34], cf. [32]]. The use of information derived from a cast shadow would have emerged at a later stage in both human evolution and development. To confirm this speculation, empirical studies with nonhuman primates should be conducted using a comparative-developmental perspective.

## Methods

### Participants

Four chimpanzees, two adult females (25-30 years old) and two young females (4-6 years old), participated in the present experiments. The young chimpanzees were raised by their biological mothers. All participants live in a social group of 14 individuals in an indoor and an environmentally enriched outdoor compound (770 m^2^) at the Primate Research Institute, Kyoto University (KUPRI), Japan. The young chimpanzees had participated in various kinds of perceptual-cognitive experiments since they were neonates [27,35-38]. They had also learned to perform computer-controlled tasks, including visual search tasks, at about one year of age [39,40]. Adult chimpanzees also had extensive experience with computer-controlled experiments [e.g., [23,24,41]]. Both of the adults had already learned to perform visual search tasks [42,43]. Chimpanzees were not deprived of food or water during the study, and no invasive treatments or special restraints were used in the present study. The care and use of the chimpanzees adhered to the 2002 version of the Guide for the Care and Use of Laboratory Primates issued by the KUPRI, which is compatible with the guidelines issued by the National Institutes of Health in the United States of America. The research design was approved by the Animal Welfare and Animal Care Committee of the KUPRI and by the Animal Research Committee of Kyoto University. All procedures adhered to the Japanese "Act on Welfare and Management of Animals."

In Experiment 2, eight human adult females with normal or corrected-to-normal visual acuity (22 years of age on average) participated as volunteers. All human participants provided informed consent for their participation in the current experiment, and they were tested in the same settings used for the chimpanzees after these were cleaned.

### Experimental Setting

Experiments were conducted in an experimental booth (1.8 × 2.15 × 1.75 m) in the experimental room adjacent to the chimpanzee facility. Each chimpanzee came to the booth via an overhead walkway connecting the facility and the booth. A 17-inch LCD monitor (1280 × 1024 pixels, pixel size: 0.264 mm × 0.264 mm) with a touch panel was installed on the wall of the booth (see Figure 2). Viewing distance was approximately 40 cm. The food reward was delivered by the universal feeder. All equipment and experimental events were controlled by the PC.

### General Procedure

Figure 2 shows an example of a trial performed by one young female chimpanzee. Each trial began with the presentation of a gray square (2.6 cm × 2.6 cm) at the bottom center of the monitor. When the chimpanzee touched this once, a search display appeared. The search display contained one target and several distractors. Each stimulus was presented at a predetermined position on a 4 × 3 virtual matrix. The configuration of the stimuli was randomly changed from trial to trial. If the chimpanzee touched the target, the search display disappeared, followed by the presentation of a chime and food reward (a small piece of apple or raisin). If she touched one of the distractors, the search display also disappeared, but the buzzer sound was presented as error feedback. Response time was defined as the time from the onset of the search display to the chimpanzee's touch of the stimulus. Following an error trial, we used a modified version of a correction procedure in which only the target was presented (correction trials). This procedure was used to prevent inappropriate runs of error trials. The intertrial interval was 2 sec.

### Experiment 1

#### Stimuli

In Experiment 1, we used so-called "Pacman" stimuli. The Pacman (or circle) was 2.6 cm in diameter and colored blue, whereas the a red square was used as "occluder" (1.3 cm × 1.3 cm). The color of the background was white. Combining these two elements, we prepared seven types of stimuli (see Figure 3A): (1) Pacman without square, (2) blue circle without square, (3) Pacman with square located on the side opposite Pacman's mouth, (4) circle with square without occluding, (5) Pacman with square located on the same side as Pacman's mouth, (6) Pacman with square at the small gap located 3 mm from Pacman's mouth, and (7) circle with occluding square. Stimuli (1), (3), (5), (6), and (7) were used as targets, and stimuli (2) and (4) were used as distractors. Stimuli (1) and (2) were presented only in the preliminary training.

#### Procedure

Although each of the chimpanzees had extensive experience with this kind of visual search task, we provided preliminary training sessions before initiating the experimental sessions. The preliminary training involved two conditions, as shown in Figure 3A: the no-square condition (target: stimulus 1) and the non-occluding square condition (target: stimulus 3). Each chimpanzee initially participated in six sessions under the no-square condition and subsequently engaged in six sessions under the non-occluding square condition. Each session consisted of 36 trials. The set size (i.e., the number of stimuli in the display) varied among four, eight, and 12 in one session (12 trials for each set size).

After pretraining, each chimpanzee participated in test sessions. Test sessions included three distance relationships between Pacman and the square, (i.e., control, gap, and occluded) in which stimulus 5, 6, or 7 appeared as the target; these were presented equally and in random order (Figure 3A). Each session consisted of 108 trials. As in the preliminary sessions, the set size varied among four, eight, and 12.

### Experiment 2

#### Stimuli

In Experiment 2, Pacman and the square were the same size as in Experiment 1, but the color of Pacman (and the circle) was changed to green. The background color was white, and we added black cast shadows to Pacman or the square for some stimuli. As shown in Figure 4, combining those elements yielded various types of stimuli differing according to the presence or absence of the cast shadows (shadow and no-shadow), the distance between Pacman and the square (control, gap, and occluded), and the spatial relationship between Pacman and the square (Pacman-shadow condition, under which Pacman cast the shadow on the square, and square-shadow condition, under which the square cast the shadow on the circle). The stimuli used in the occluded conditions under the no-shadow and shadow conditions were identical. Note that the shape of the square under the Pacman-shadow/no-shadow/gap condition was the same as that under the Pacman-shadow/shadow/gap condition to hold the size of the area with red pixels constant. In Experiment 2, the gap width (i.e., width of the cast shadow) was smaller (1.3 mm) than that in Experiment 1. Note that the gap width depicted in Figure 4 is exaggerated rather than drawn to scale.

#### Procedure

The experimental procedure was the same as that followed in Experiment 1 and was the same for both chimpanzee and human participants, except that humans did not receive a food reward and correction trials after error trials.

Chimpanzee participants were shifted to Experiment 2 immediately after completing Experiment 1. Before test sessions, they received a single 32-trial pretraining session in which the target was a Pacman with a square located at the side opposite to Pacman's "mouth." The background color was white as in Experiment 1. The set size was fixed at eight. Human participants received a single pretraining session, which was the same as that for chimpanzees except for the number of trials (= 16).

After preliminary training, each chimpanzee participated in sessions under the square-shadow and Pacman-shadow conditions. The type of session was alternated, and each type was repeated five times. The set size (the number of stimuli in the display) was set at eight. The presence/absence of shadows and the three types of distances involving Pacman and the square were equally but randomly distributed in each session. Each session consisted of 48 trials. For human participants, each type of session was repeated twice, and participants were presented with only the chime and not a food reward following correct responses.

### Data analyses

We analyzed the accuracy and response-time data for the test sessions using general linear mixed models (using SPSS 14.0J) in which the Pacman-square distance (control, gap, and occluded) and number of stimuli in Experiment 1, and the Pacman-square distance (control, gap, and occluded) and presence/absence of shadows in Experiment 2 represented fixed effects, and the participants and the sessions nested within participants served as random effects. In Experiment 2, separate analyses were conducted for square-shadow and Pacman-shadow conditions for each species. The level of statistical significance was set at 0.05.

## Competing interests

The authors declare that they have no competing interests.

## Authors' contributions

MT conceived of the study and designed the experiments; MT and TI conducted the experiments, analyzed the data, and wrote the manuscript. Both authors read and approved the final manuscript.
